# Supplementary material for: Ciliate mitoribosome illuminates evolutionary steps of mitochondrial translation
Source: eLife. 2020 Jun 18;9:e59264. doi: 10.7554/eLife.59264 (PMC7326499; doi:10.7554/eLife.59264)
Supplement: Supplementary file 1. [file elife-59264-supp1.pdf]

Supplementary File 1. Cryo-EM data collection, refinement and validation statistics

	Consensus map	LSU mask	SSU mask	CP mask	L7/L12 mask	Head mask	Back protuberance mask
**Data collection and processing**							
Magnification	130000x	130000x	130000x	130000x	130000x	130000x	130000x
Voltage (kV)	300	300	300	300	300	300	300
Electron exposure (e–/Å^2)	30	30	30	30	30	30	30
Defocus range (μm)	-0.4 ~ -3.0	-0.4 ~ -3.0	-0.4 ~ -3.0	-0.4 ~ -3.0	-0.4 ~ -3.0	-0.4 ~ -3.0	-0.4 ~ -3.0
Pixel size (Å)	1.07	1.07	1.07	1.07	1.07	1.07	1.07
Symmetry imposed	C1	C1	C1	C1	C1	C1	C1
Initial particle images (no.)	450250	450250	450250	450250	450250	450250	450250
Final particle images (no.)	99380	99380	99380	99380	99380	99380	99380
Map resolution (Å)	3.67	3.38	3.61	3.61	3.48	3.30	3.61
FSC threshold 0.143							
Map resolution range (Å)	3.4~10	3.0~10	3.2~10	3.4~10	3.2~10	2.9~8	3.3~10
**Refinement**							
Map sharpening *B* factor (Å^2)	-70						
Model composition							
Non-hydrogen atoms	282876						
Protein residues	23994						
Ligands	398						
B factors (Å^2)							
Protein	98						
Nucleotide	83						
Ligand	143						
R.m.s. deviations							
Bond lengths (Å)	0.009						
Bond angles (°)	0.948						
Validation							
MolProbity score	1.52						
Clashscore	3.79						
Poor rotamers (%)	0.01						
Ramachandran plot							
Favored (%)	94.98						
Allowed (%)	4.99						
Disallowed (%)	0.03						
