# Supplementary material for: Ciliate mitoribosome illuminates evolutionary steps of mitochondrial translation
Source: eLife. 2020 Jun 18;9:e59264. doi: 10.7554/eLife.59264 (PMC7326499; doi:10.7554/eLife.59264)
Supplement: Supplementary file 2. [file elife-59264-supp2.pdf]

Supplementary File 2: Summary of the mitoribosomal proteins.

Alias	Chain ID	Uniprot Name	ID	Full Size	Modeled Residues	Comment
LSU rRNA 1	Aa		AF396436.1: 47156-47433	287	1-278	
LSU rRNA 2	Ab		AF396436.1: 44750-47063	2314	279-1317, 1324-1816, 1886-2237	L1 stalk not modeled
uL2m	Ac	Ribosomal protein L2	Q951B6	262	1-262	
uL3m	Ad	50S ribosomal protein L3	Q22HG3	439	32-59, 85-439	
uL4m	Ae	50S ribosomal protein L4	I7MGP2	358	22-359	
uL5m	Af	Ymf69	Q951A2	68	1-68	
uL6m	Ag	Ymf60	Q951A9	179	1-176	
bL7/12m	Ah, Ai, Aj, Ak	Ribosomal protein L7/L12 Carboxy-terminal domain	Q24IM4	280	1-117: 1-61: 1-75: 12-116	Four chains
bL9m	Al	Ribosomal protein L9, Amine-terminal domain	Q24C33	223	12-64	Majority missing due to flexible L1 stalk
bL10m	Am	Ymf74	Q951B5	157	(-8)-157	Additional amino acids at N-terminus modeled as UNK with negative numbers
uL11m	An	Ribosomal protein L11, Amine-terminal domain	W7XH77	158	1-158	
uL13m	Ao	50S ribosomal protein L13	W7X626	391	28-357	
uL14m	Ap	Ribosomal protein L14	Q950Y1	119	1-119	
uL15m	Aq	Ribosomal protein L15, putative	I7MF78	305	33-305	
uL16m	Ar	Ribosomal protein L16	Q951A0	143	1-143	
bL17m	As	50S ribosomal protein L17	A4VE95	237	2-237	
bL19m	At	Uncharacterized protein	I7M2I9	242	1-183	
bL20m	Au	50S ribosomal protein L20	Q235I3	170	2-170	
bL21m	Av	50S ribosomal protein L21	I7LVD3	235	30-235	
uL22m	Aw	Ribosomal protein L22/L17e	Q23CT9	364	23-364	
uL23m	Ax	Ribosomal protein L23 putative	Q22EY1	138	6-138	
uL24m	Ay	50S ribosomal protein L24	Q24C34	234	45-234	
bL25m	Az	Uncharacterized protein	I7LVZ3	321	26-262	Contains two domains as observed in alphaproteobacteria.
bL27m	AA	50S ribosomal protein L27	I7M068	237	38-213	
bL28m	AB	Ribosomal protein L28	I7LU83	289	23-286	
uL29m	AC	39-S ribosomal protein L47	Q22W36	307	21-304	Sequence missannotated as mL47
bL32m	AD				1-36	No identified sequence
bL33m	AE	50S ribosomal protein L33	Q22L35	64	6-62	
bL35m	AF	Uncharacterized protein	I7M803	164	50-164	
bL36m	AG	Ribosomal protein	W7XH61	93	1-38	Zn binding
mL40	AH	Uncharacterized protein	I7LT48	199	23-199	
mL41	AI	Ribosomal protein L27	I7MG40	155	11-132	Sequence missannotated as bL27m
mL43	AJ	Ribosomal protein L51/S25/CI-B8 domain	Q22RG0	179	2-179	
mL46	AK	Uncharacterized protein	I7LWM6	309	66-309	
mL49	AL	Large subunit Ribosomal protein	I7MAF1	145	1-145	
mL53	AM	Uncharacterized protein	Q22KC0	184	59-184	
mL54	AN	Uncharacterized protein	I7MI39	158	40-135	
mL64	AO	Uncharacterized protein	Q23ZH7	155	1-120	

Alias	Chain ID	Uniprot Name	ID	Full Size	Modeled Residues	Comment
mL101	AP	Uncharacterized protein	I7M3V9	386	23-386	Helical repeat protein binding partially unfolded H79
mL102	AQ	Uncharacterized protein	W7WYR3	109	60-109	
mL103	AR	Uncharacterized protein	I7MKV5	348	62-335	
mL104	AS	Uncharacterized protein	I7LTP6	763	24-97, 111-764	Helical repeat protein binding H42-ES1
mL105	AT	Signal peptide-binding Domain protein	W7XFS0	299	134-299	Homolog of bacterial SRP binding protein Ffh
mL106	AU	Uncharacterized protein	Q23Q81	499	21-49	Helical repeat protein binding LSU 3'-ES1
mL107	AV	Iron-binding zinc f nger CDGSH Type protein	Q22HE3	160	22-152	FES cluster binding CDGSH motif protein

Alias	Chain ID	Uniprot Name	ID	Full Size	Modeled Residues	Comment
SSU rRNA 1	Ba		AF396436.1: 32623-32833	211	14-209	
SSU rRNA 2	Bb		AF396436.1: 32895-34289	1395	218-1602	
us2m	Bc	Ymf73	Q950X8	159	1-159	
uS3m	Bd	Ymf64	Q951A8	330	1-330	Constitutes the CTD of bacterial uS3. Assembles together with mS31 and mS92 to produce a full S3 protein.
uS4m	Be	Ymf76	Q951C0	405	1-405	
uS5m	Bf	30S ribosomal protein S5	I7M4M7	351	16-352	
bS6m	Bg	Ribosomal protein S6	I7MHS2	141	2-137	
uS7m	Bh	Ymf63	Q951A4	276	1-276	
uS9m	Bi	Ribosomal protein S9	I7M4A2	737	23-184, 256-737	
uS10m	Bj	Ymf59	Q951A1	152	1-152	
uS11m	Bk	Ymf61	Q950Z8	238	13-238	
uS12m	Bl	Ribosomal protein S12	Q951B3	276	5-133	
uS13m	Bm	Ribosomal protein S13	Q951B9	101	3-19, 213-230, 240-248	
uS14m	Bn	Ribosomal protein S14	Q951B0	196	1-101	
uS15m	Bo	30S ribosomal protein S15	Q22WF3	133	18-196	
bS16m	Bp	30S ribosomal protein S16	I7M3F6	437	1-412	
uS17m	Bq	30S ribosomal protein S17	I7M6C7	205	2-112, 119-183	
bS18m	Br	Ribosomal protein S18	Q23K82	549	27-549	
uS19m	Bs	Ribosomal protein S19	Q951B7	98	1-87	
bS21m	Bt	Uncharacterized protein	Q22BA0	102	1-102	
mS23	Bu	Uncharacterized protein	Q23YQ0	675	1-217, 227-448, 457-583	
mS26	Bv	Uncharacterized protein	I7M0P0	579	25-334, 425-589	
mS29	Bw	Ribosomal death-associated protein	Q22RT0	703	22-38, 49-110, 121-705	ATP binding, additional domain compared to *H. sapiens*
mS31	Bx	Uncharacterized protein	Q23UD3	719	182-387, 396-719	Assembles the uS3m NTD together with uS3m and mS92
mS33	By	Uncharacterized protein	I7LW04	132	2-108	
mS34	Bz	Uncharacterized protein	I7LWU8	147	10-132	
mS35	BA	Ribosomal subunit protein	Q23K64	149	12-149	
mS37	BB	Uncharacterized protein	I7MMM6	112	3-112	
mS38	BC				3-37	No identified sequence
mS41	BD	IGR motif protein	Q22FZ4	130	24-130	
mS45	BE	Uncharacterized protein	Q23DN6	464	18-464	

Alias	Chain ID	Uniprot Name	ID	Full Size	Modeled Residues	Comment
mS75	BF				1-23	Charge compensating peptide in SSU head, no identified sequence
mS76	BG	Uncharacterized protein	Q24G80	182	19-182	
mS77	BH				1-63	No identified sequence, possible fragment
mS47	BI	Enoyl-CoA Hydratase/isomerase	Q22P47	1451	24-1019, 1094-1510	rRNA binding protein domain with putative lipase fold interacting with 5' SSU rRNA. The remaining protein sequence has minimal tertiary structure but forms interactions with most proteins in the back protuberance.
mS78	BJ	Uncharacterized protein	I7M7B1	1539	25-1539	Large scaffolding protein connecting SSU head, back protuberance and body extension.<br>Contains a helical repeat domain not binding rRNA
mS79	BK	Uncharacterized protein	Q23DG3	267	1-267	
mS80	BL	SelR domain protein	Q22W57	310	123-303	Zn binding methionine oxidoreductase
mS81	BM	Uncharacterized protein	Q24E31	355	72-335	
mS82	BN	Uncharacterized protein	Q22GF7	283	10-283	
mS83	BO	Uncharacterized protein	Q22N51	1338	107-285	Helical repeat protein in back protuberance. Continues with extremely poor density. Density suggests additional flexible domains
mS84	BP				1-100	No identified sequence
mS85	BQ	Uncharacterized protein	Q22UP3	1032	39-553, 578-684	
mS86	BR	Iron donor protein CyaY	Q240K3	143	1-143	Fratraxin like fold, no Fe bound.
mS87	BS	Uncharacterized protein	A4VCP7	1086	646-841, 850-1086	
mS88	BT				1-299	No identified sequence
mS89	BU	Uncharacterized protein	Q22EB6	439	29-337	
mS90	BV	Uncharacterized protein (Fragment)	Q24HL0	401	79-422	
mS91	BW				1-286	No identified sequence. Helical repeat protein in back protuberance.
mS92	BX	Ribosomal protein S3	Q951B8	151	1-62, 75-118, 125-151	Part of uS3m together with mS31
mS93	BY	mTERF protein	I7LUK3	411	17-387	Mitochondrial transcription termination factor MTERF4

Components of the *T.thermophila* mitochondrial ribosome. Conserved proteins in blue, shared mitoribosomal proteins in red, specific mitoribosomal proteins in yellow. ID corresponds to Uniprot identifier for proteins and GenBank accession number and range for rRNA.
